# Crosstalk between Lysosomes and Mitochondria in Parkinson's Disease

**DOI:** 10.3389/fcell.2017.00110

**Published:** 2017-12-12

**Authors:** Nicoletta Plotegher, Michael R. Duchen

**Affiliations:** Department of Cell and Developmental Biology and UCL Consortium for Mitochondrial Research, University College London, London, United Kingdom

**Keywords:** alpha-synuclein, LRRK2, autophagy, mitophagy, lysosomes, mitochondria, Parkinson's disease, neurodegeneration

## Abstract

Parkinson's disease (PD) is the most common motor neurodegenerative disorder. In most cases the cause of the disease is unknown, while in about 10% of subjects, it is associated with mutations in a number of different genes. Several different mutations in 15 genes have been identified as causing familial forms of the disease, while many others have been identified as risk factors. A striking number of these genes are either involved in the regulation of mitochondrial function or of endo-lysosomal pathways. Mutations affecting one of these two pathways are often coupled with defects in the other pathway, suggesting a crosstalk between them. Moreover, PD-linked mutations in genes encoding proteins with other functions are frequently associated with defects in mitochondrial and/or autophagy/lysosomal function as a secondary effect. Even toxins that impair mitochondrial function and cause parkinsonian phenotypes, such as rotenone, also impair lysosomal function. In this review, we explore the reciprocal relationship between mitochondrial and lysosomal pathways in PD. We will discuss the impact of mitochondrial dysfunction on the lysosomal compartment and of endo-lysosomal defects on mitochondrial function, and explore the roles of both causative genes and genes that are risk factors for PD. Understanding the pathways that govern these interactions should help to define a framework to understand the roles and mechanisms of mitochondrial and lysosomal miscommunication in the pathophysiology of PD.

## Introduction

Parkinson's disease (PD) is a multifactorial and genetically heterogeneous neurodegenerative disorder, characterized by bradykinesia, resting tremor, postural instability, and muscle rigidity. Together with motor symptoms, more variable non-motor symptoms may develop, including anosmia, sleep disorders, depression and, with disease progression, dementia. The primary neurodegenerative pathology features the loss of dopaminergic neurons in the s*ubstantia nigra pars compacta*, while the main histopathological hallmark seen at post mortem is the presence in the surviving neurons of protein aggregates, known as Lewy bodies (LB).

The cause of PD in the vast majority of subjects is unknown—referred to as “sporadic”—or associated with exposure to environmental toxins (Tanner et al., 2011). In about 10% of subjects, it is caused by monogenic mutations and 15 causative genes have so far been identified associated with familial disease, frequently of early onset (Table 1). Genome-wide association studies have revealed mutations in many other genes that increase disease risk. Many of the causative or risk factor genes for PD show an association with mitochondrial quality control pathways, ranging from mitochondrial proteins to proteins that regulate endo-lysosomal function (Verstraeten et al., 2015).

**Table 1:** Overview of PD causative genes, their function(s), the clinical features of the PD forms associated with their mutations and the involvement of mitochondrial and/or endo-lysosomal dysfunction.

**Gene**	**Primary function**	**Endo-lysosomal defects**	**Mitochondrial defects**	**Mode of inheritance**	**PD form and pathology**
SNCA	Synaptic vesicles	Yes	Yes	AD	EO or LO; alpha-synuclein accumulation
parkin	Mitophagy	NA	Yes	AR	EO; occasional alpha-synuclein accumulation
DJ-1	Oxidative stress; chaperone	Yes	Yes	AR	EO
LRRK2	Endo-lysosomal trafficking and function	Yes	Yes	AD	LO; alpha-synuclein, tau, and TDP-43 accumulation
PINK1	Mitophagy	Yes	Yes	AR	EO; occasional alpha-synuclein accumulation
ATP13A2	Lysosomal ATPase, cation homeostasis	Yes	Yes	AR	JO; iron accumulation
FBXO7	Adaptor protein in SCF ubiquitin E3 ligase	NA	Yes	AR	JO
PLA2G6	A2 phospholipase (phosphatidylcholine)	NA	Yes	AR	JO; iron accumulation
VPS35	Retromer complex; protein trafficking	Yes	Yes	AD	LO
EIF4G1	Recruitment of mRNA to the ribosome	NA	NA	AD	LO; alpha-synuclein, tau and Aβ accumulation
DNAJC6	Clathrin-mediated endocytosis	NA	Yes	AR	JO
ATP6AP2	Vacuolar ATPase component, lysosomal pH	Yes	Likely	X linked	JO or EO; tau accumulation
COQ2	Coenzyme Q10 biosynthesis	NA	Yes	AR	LO; alpha-synuclein accumulation
SYNJ1	Clathrin coated vesicles disassembly	NA	NA	AR	JO
DNAJC13	Retromer-mediated endosomal protein sorting	Yes	NA	AD	LO; alpha-synuclein accumulation

*EO, early onset; LO, late onset; JO, juvenile onset; AR, autosomal recessive; AD, autosomal dominant; NA, unknown*.

Interestingly, the familial forms of the disease associated with mutations of proteins involved in the autophagic-lysosomal pathway, often show mitochondrial defects (Gusdon et al., 2012; Ramonet et al., 2012; Tang et al., 2015; Wang et al., 2015). Moreover, alpha-synuclein (aS) aggregation, as well as LRRK2 mutations, which cause PD, can be both involved in dysregulation of autophagic and endo-lysosomal pathways through different mechanisms, and are also associated with mitochondrial dysfunction (Niu et al., 2012; Papkovskaia et al., 2012; Nakamura, 2013; Yang et al., 2014). On the other hand, mitochondrial complex I inhibition induced by rotenone [a toxin that cause parkinsonian phenotype (Tanner et al., 2011)] alters the expression of lysosomal genes (Fernández-Mosquera et al., 2017). To be noted is the observation that mitochondrial complex I deficiencies were associated with sporadic forms of PD, making this defect one of the more common features of the pathophysiology of PD (Schapira et al., 1990).

More generally, several studies have shown that lysosomal dysfunction impacts on mitochondria by impairing mitophagy and possibly by changes in other signaling pathways. These mechanisms appear to play a significant role in the pathophysiology of other diseases, especially in lysosomal storage disorders (LSD) (Plotegher and Duchen, 2017). Remarkably, mitochondrial defects have also been shown to impact on lysosomal function (Diogo et al., 2017), suggesting a complex reciprocal relationship between these two compartments (Raimundo et al., 2016).

In this review, we will discuss some of the genes whose mutations are associated with PD and will focus on the crosstalk between mitochondria and endo-lysosomal pathway in the pathophysiology of genetic PD.

## From mitochondria to lysosomes in PD: are the power houses of the cell clogging their recycle bins?

Mutations in parkin, PINK1 and DJ-1 cause autosomal recessive parkinsonism, and have come to represent the archetype of PD associated with impaired mitophagy. Both parkin and PINK1 encode proteins involved in the regulation of mitophagy. The clearance of dysfunctional mitochondria can be initiated through several different pathways, among which the parkin/PINK1 pathway is one of the most extensively characterized. The loss of mitochondrial membrane potential leads to PINK1 accumulation on the outer mitochondrial membrane. PINK1 recruits the E3 ubiquitin ligase parkin to the mitochondria (Narendra et al., 2010), and the activation of parkin leads to the ubiquitination of mitochondrial membranes, initiating the removal of dysfunctional organelles (Narendra et al., 2008).

While mutations in PINK1 and parkin apparently affect mitochondrial function because they permit the accumulation of dysfunctional mitochondria, it was also shown that parkin-deficiency in PD patient fibroblasts causes defects in the function of retromer, a trimeric cargo-recognition protein complex responsible for protein trafficking in the endosomal compartment (Song et al., 2016). Moreover, PINK1 depletion inhibits lysosomal function and induces the enlargement of the vacuolar compartment. Defects in the lysosomal compartment also occurred in response to inhibition of the mitochondrial ATP-synthase using oligomycin (Demers-Lamarche et al., 2016) and in T cells from mice with knockout of *Tfam*, the major transcription factor in mitochondrial biogenesis (Baixauli et al., 2015).

Of special relevance for PD is the recent characterization of the effects of rotenone treatment on lysosomal biogenesis. Rotenone is a pesticide that inhibits complex I of the mitochondrial respiratory chain. It induces a parkinsonian phenotype in animal models (Liu et al., 2015) and epidemiological studies suggest that environmental exposure to rotenone may increase the risk of PD in humans (Tanner et al., 2011). Acute exposure of mouse embryonic fibroblasts to rotenone caused a rapid increase in the transcript level of some lysosomal genes, while chronic treatment induced a marked decrease in the expression of these same genes (Fernández-Mosquera et al., 2017). Considering the defects in complex I function described in sporadic PD, it seems important to understand how this affects lysosomal function in these forms of PD.

**Figure 1:**
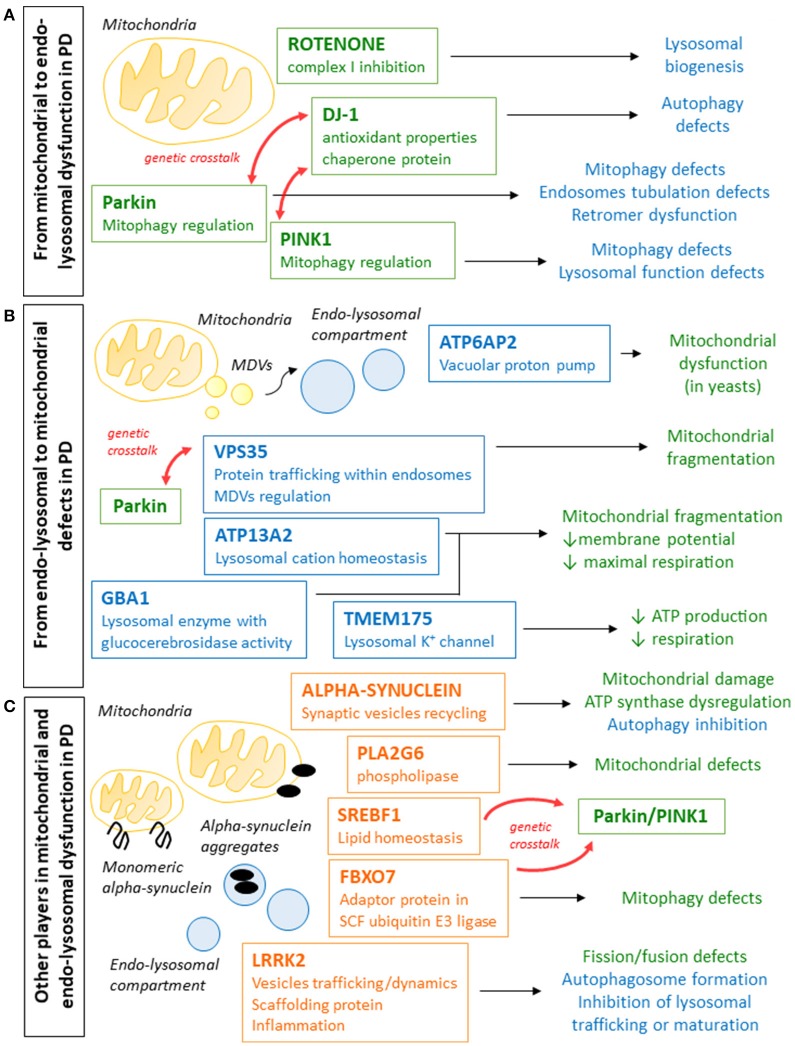
Schematic representation of the different ways in which genes whose mutations are involved in PD pathogenesis cause defects in mitochondrial and endo-lysosomal compartments. **(A)** Parkin, PINK1, and DJ-1 mutations cause PD and are all involved in the regulation of mitophagy and mitochondrial quality control and mitophagy regulation, but can also lead to endo-lysosomal pathway defects and autophagy impairment. Rotenone, which inhibits mitochondrial complex I and was associated to parkinsonism in animal models and in epidemiological studies, also impacts on lysosomal compartment. **(B)** Genes whose mutations cause PD (VPS35, ATP6AP2, and ATP13A2) or increase the risk (GBA1 and TMEM175) are often involved in endo-lysosomal pathway function. Defects in these pathway can have effects on the mitochondrial function, leading reduced membrane potential, defective mitochondrial respiration and reduced ATP production. **(C)** Mutations in alpha-synuclein and LRRK2, whose functions have not been fully elucidated yet, cause autosomal dominant PD and were shown to affect both mitochondrial and endo-lysosomal function. Other genes whose mutations were associated to PD (PLA2G6, FBXO7, and SBERF1) and whose functions are not directly related to mitochondria or lysosomal compartment, were shown to affect mitochondrial clearance and to interact with other genes relevant to PD, and it cannot be excluded that the endo-lysosomal pathway play a role also in these cases.

The PD-related protein DJ-1 localizes to mitochondria and seems to act as an antioxidant and chaperone, although its specific role remains controversial (Junn et al., 2009; Girotto et al., 2014). DJ-1 is involved in both mitochondrial function and autophagy (Thomas et al., 2011): DJ-1 silencing in the M17 neuroblastoma cell line caused a reduction of mitochondrial membrane potential, mitochondrial fragmentation and accumulation of autophagy markers. Overexpression of parkin or PINK1 proteins rescued this phenotype. Also DJ-1 knockout flies showed defects in mitochondrial respiration and reduced ATP production. Interestingly, DJ-1 overexpression can rescue the disease phenotype in PINK1 deficient flies, but not in *parkin*^−/−^ flies (Hao et al., 2010). These results further suggest that DJ-1 plays a role in the control of mitochondrial homeostasis, likely involving the PINK1/parkin pathway. DJ-1 deficiencies also impact on the autophagy pathway, with a less clear mechanism that may depend on mitochondrial defects (Mccoy and Cookson, 2017). This suggest that jeopardizing mitochondrial function at any level (quality control, dynamics, or respiration) can impact on the function of the lysosomal compartment in PD (Figure 1A).

## From the endo-lysosomal pathway to mitochondria in familial PD: when garbage stops energy production

In the last few years, extensive efforts led to the identification of novel genes implicated in the pathogenesis of PD. Among these genes, it is striking that many encode proteins involved in endo-lysosomal trafficking and function. Defects in this pathway can negatively affect mitochondrial function and can interfere with the removal of defective mitochondria, also through impaired autophagy (Figure 1B).

Vacuolar protein sorting-associated protein 35 (Vps35) is part of the retromer, which regulates protein trafficking within the endosomal compartment, and the pathogenic D620N mutation in Vps35 causes alterations in endosomes and trafficking defects (for example, it disrupts the trafficking of cathepsin D, a lysosomal protease) (Follett et al., 2013). Cathepsin D trafficking defects may contribute to lysosomal deficiencies, but most studies have shown that the main victims of pathogenic mutation of Vps35 seem to be mitochondria.

In fact, it was suggested that Vps35 is responsible for mediating transport of mitochondrial derived vesicles (MDVs) from mitochondria to other cellular compartments (Soubannier et al., 2012), therefore being involved in mitochondria quality control. Moreover, Tang et al. showed that in mouse dopaminergic neurons, depletion of Vps35 promotes degradation of Mitochondrial E3 Ubiquitin Protein Ligase 1 (MUL1), thus reducing Mitofusin 2 (Mfn2) stabilization and impeding mitochondrial fusion (Tang et al., 2015). Mitochondrial fragmentation was also observed in cultured neurons, in mice and in fibroblasts carrying the D620N Vps35 mutation (Tang et al., 2015; Wang et al., 2015). The mechanism causing these defects was associated with the increased turnover of the mitochondrial protein dynamin-like protein 1 (DLP1) through increased trafficking of MDVs to lysosomes for degradation.

Vps35 was also linked to the parkin pathway. Malik et al. showed that Vps35 and parkin pathways may interact in flies (Malik et al., 2015). In particular, flies heterozygous for both *vps35/*+;*parkin/*+ were more sensitive to the herbicide paraquat [which, similarly to rotenone, is a toxin linked to PD (Tanner et al., 2011)] and presented degeneration of dopaminergic neurons. Moreover, Vps35 overexpression in *parkin*-mutant fly models rescued many of the phenotypes, but the same was not true for *pink1*-mutant flies.

Another gene whose mutations cause an atypical form of PD, termed the Kufor-Rakeb syndrome, but also a complicated form of hereditary spastic paraplegia (Estrada-Cuzcano et al., 2017) is ATP13A2. The protein encoded by this gene is a lysosomal P-type ATPase that seems to be involved in cation homeostasis (Ramonet et al., 2012). Mutations of ATP13A2 are associated with marked changes in lysosomal and mitochondrial function. Modulation of ATP13A2 expression decrease cytosolic calcium levels, while silencing ATP13A2 induces mitochondrial fragmentation (Ramonet et al., 2012). In fibroblasts from patients carrying mutations in ATP13A2, mitochondrial maximal respiratory rate and membrane potential were reduced (Grünewald et al., 2012). Gusdon et al. showed that ATP13A2 knockout in neurons and SHSY5Y cells increased mitochondrial mass, suggesting decreased turnover (Gusdon et al., 2012), likely associated with impaired autophagic flux, causing failure of clearance of defective mitochondria. However, we cannot exclude that other pathways link ATP13A2 deficiencies to mitochondrial dysfunction.

Mutations of the ATPase H^+^ Transporting Accessory Protein 2 (ATP6AP2) gene cause a rare X-linked form of parkinsonism with spasticity (Korvatska et al., 2013). ATP6AP2 transmembrane protein is an accessory component of the vacuolar ATPase, essential to keep lysosomal pH acidic. ATP6AP2 deficiencies cause the accumulation of autophagosomes and autophagolysosomes, and p62, suggesting impairment in autophagy and lysosomal clearance (Korvatska et al., 2013). A link between alkalinization of acidic vacuoles and mitochondrial dysfunction was established in yeast, and has aso been described in mammalian cells (Hughes and Gottschling, 2012). It was recently shown that lysosomal pH alkalinization impairs autophagy (Trudeau et al., 2016), while lysosome re-acidification rescued the phenotype.

More studies are needed to understand whether impairment of the autophagy-lysosomal pathway is associated with mitochondrial defects because they permit the accumulation of defective mitochondria through failed mitophagy or whether other pathways are involved.

## Roles of alpha-synuclein and LRRK2 in endo-lysosomal pathway defects and mitochondrial dysfunction

aS is localized at the presynaptic terminal in the mammalian brain and is involved in synaptic vesicle recycling and docking (Burré et al., 2010). Several single point mutations and gene multiplications cause autosomal dominant PD. Moreover, aS fibrils are the main constituent of Lewy Bodies and aS oligomers and aggregates play a key role in neuronal death (Plotegher et al., 2014b).

The accumulation of aS oligomers and fibrils was shown to be involved in both impaired impairment (Xilouri et al., 2016) and mitochondrial dysfunction (Nakamura et al., 2011; Plotegher et al., 2014a; Pozo Devoto et al., 2017). Moreover, impaired autophagy itself was shown to be associated with aS accumulation, further amplifying this detrimental mechanism (Xilouri et al., 2016) and likely affecting also the removal of dysfunctional mitochondria.

Mutations of LRRK2, a large kinase that also shows GTPase activity, account for some 40% of genetic cases of PD. LRRK2 has been shown to play a role in many different pathways, including the endo-lysosomal (Roosen and Cookson, 2016), but its exact functions are still unclear. LRRK2 interacts with many proteins in the endo-lysosomal compartment, such as proteins from the Rab-family, and it can play a role in both autophagosome formation but also in the maturation or in the trafficking of lysosomes.

LRRK2 mutations associated with PD were shown to alter mitochondrial fusion/fission by interfering with the mitochondrial fission factor DLP1 (Niu et al., 2012; Yang et al., 2014). Interestingly, DLP1 mediated defects in mitochondrial dynamics were also seen in association with Vps35 mutations (Wang et al., 2015). Another study suggested that LRRK2 mutations impact on mitochondrial function by causing reduced membrane potential and ATP production (Papkovskaia et al., 2012), which could be associated with defective fusion and fission reported by others.

In both aS and LRRK2-associated PD, both mitochondria and lysosomal compartments are affected, but it is still unclear in which organelles the damage starts and how it is then extended to affect the other (Figure 1C). One idea is that an impaired autophagic-lysosomal pathway associated with LRRK2 mutations or due to aS clumps causes the accumulation of dysfunctional mitochondria; another is that mitochondrial damage induced by aS aggregates or LRRK2 defects impact on lysosomal function and biogenesis, as observed for other models showing defective mitochondria. It is possible that both pathways may be activated at the same time, making it even more difficult to understand the pathophysiological cascade.

## New players, key links to mitochondrial defects?

Mutations of several other genes have been shown to cause PD and even if the pathological mechanism(s) are not yet well-understood, a few examples may provide new hints to illuminate the interplay between the endo-lysosomal compartment and mitochondrial network in the pathogenesis of PD.

PLA2G6 mutations have been associated with a variety of neurological disorders collectively termed PLA2G6-associated neurodegeneration (PLAN) among which there is an autosomal recessive form of dystonia-parkinsonism (Paisan-Ruiz et al., 2009). The gene encodes a calcium-independent phospholipase, which localizes to mitochondria and generates free fatty acids by catalyzing the hydrolysis of glycerophospholipids (Gadd et al., 2006). For infantile neuroaxonal dystrophy, which is one of the PLAN disorders, Beck et al. have shown that mitochondria were involved in the neurodegeneration (Beck et al., 2011), suggesting that axonal degeneration is a consequence of the presence of abnormal mitochondria following axonal transportation. More recently, in flies in which the *Drosophila* homolog of PLA2G6 (iPLA2-VIA) was knocked out and in patient fibroblasts carrying PLA2G6 mutations, mitochondrial function was severely compromised, with reduced mitochondrial membrane potential, respiration, and ATP production (Bartolome et al., 2015).

The gene *FBXO7* encodes an adaptor protein in Skp-Cullin-F-box (SCF) ubiquitin E3 ligase complex responsible for mediating its ubiquitination by the SCF E3 ligase. Mutations in this gene cause a Parkinsonian-pyramidal syndrome. FBXO7 interacts with the ubiquitin E3 ligase parkin (Burchell et al., 2013) and participates in the recruitment of parkin at the mitochondrial membrane following depolarization to initiate mitophagy. Moreover, as for Vps35, overexpression of FBXO7 rescued the disease phenotype in parkin-deficient flies, but not in PINK1 mutants. It is especially interesting that Vps35 and FBXO7 act in the same way in terms of the interaction with the Parkin/PINK1 pathway although the two proteins have completely different primary functions.

How defects in the endo-lysosomal compartment contribute to the neurodegeneration in these forms of PD and how and whether this may be associated with the observed mitochondrial defects remain to be fully elucidated (Figure 1C).

## Hints from genes found to be risk factors for PD involving lysosomal-mitochondrial communication

Over 25 genes associated with an increased risk of developing PD have been identified through genome-wide association studies (Verstraeten et al., 2015). Among them, some are of particular relevance for this review. First of all, the most common genetic risk factor for PD is represented by heterozygous mutations in *GBA1*, a gene encoding for the lysosomal enzyme glucocerebrosidase (Sidransky et al., 2009). *GBA1* homozygous mutations cause the LSD known as Gaucher's disease (GD) and some GD patients and their (heterozygous, carrier) relatives show parkinsonian manifestations (Tayebi et al., 2001).

In neurons cultured from a GBA1 knockout mouse, a model for a severe neurological form of GD, autophagy was impaired upstream of the lysosomes, and at the same time mitochondrial function was profoundly compromised, with a reduced membrane potential, severely impaired respiration and mitochondrial fragmentation (Osellame et al., 2013). Mitochondrial function was also impaired in fibroblasts from GD patients (de la Mata et al., 2015). Autophagy defects have also been documented in iPSC-derived neurons from PD patients carrying GBA1 mutations (Schöndorf et al., 2014). Moreover, in many models and patients, GBA1 deficiencies were associated with aS accumulation and aggregation (Choi et al., 2011; Cullen et al., 2011; Osellame et al., 2013), establishing a solid link between the two genes and involving aS as a key element in GBA1-associated neurodegeneration in PD, likely through impairment of the autophagic-lysosomal pathway and mitochondrial dysfunction, possibly attributable to accumulation of dysfunctional mitochondria as a consequence of defective mitophagy.

Mutations in the lysosomal K^+^ channel TMEM175 (Jinn et al., 2017) also represent significant risk factors for PD. TMEM175 depletion in SHSY5Y cells was associated with increased lysosomal pH and with reduction in lysosomal degradative capacity. At the same time, mitochondrial respiration was impaired and ATP production reduced. Therefore, a primary lysosomal defect in PD impairs autophagy and mitochondrial function through a mechanism that has yet to be fully elucidated.

Mutations in the sterol regulatory element binding transcription factor 1 (SREBF1), a transcription factor necessary for lipid homeostasis, also increase the risk of developing PD. SREBF1 was also identified as one of a group of genes with a conserved role in mitophagy, favoring parkin translocation to mitochondria to initiate the process (Ivatt et al., 2014).

Overall these risk factor genes for PD point toward a dysregulation in endo-lysosomal compartment that impacts on mitochondria, or toward mitochondrial defects that signal to lysosomes affecting their function (Figure 1B). Further studies will be needed to better understand the role for these genes in sporadic PD.

## Conclusions

A remarkable number of genes associated with PD, either causing disease or increasing risk, are associated with endo-lysosomal or mitochondrial defects. However, what is more striking is the fact that a primary defect in either of the two compartments usually leads to damage of the other, suggesting a strong reciprocal relationship between them, and placing the interconnection between the two center stage in the pathogenesis of the disease.

While in the past the link mainly referred to the fact that dysfunctional autophagy also implied dysfunctional mitophagy and therefore accumulation of defective mitochondria, now new avenues are emerging, suggesting that a more complex web of signaling pathways may link the two and give rise to disease.

On the mitochondrial side, strong evidence points to a role for impaired mitochondrial function in signaling to lysosomal pathways, affecting lysosomal biogenesis and function. When this leads to lysosome defects, it can also impact the autophagy-lysosomal pathway upstream, inducing impaired autophagy.

More efforts will be needed to understand how the interplay between mitochondrial and lysosomal dysfunction play a role in PD pathogenesis, and whether these pathways represent a potential generalized therapeutic target for sporadic and familial PD.

## Author contributions

NP and MD conceived and wrote the manuscript.

### Conflict of interest statement

The authors declare that the research was conducted in the absence of any commercial or financial relationships that could be construed as a potential conflict of interest.
